# From Innovation to Impact: The CREATE Framework as a Blueprint for Large Language Model Adoption in Opioid Treatment Programs

**DOI:** 10.2196/97131

**Published:** 2026-10-07

**Authors:** Marianthi Markatou, Raktim Mukhopadhyay, Jeff Good, Yihao Tan, Arpan Dharia, Lawrence S Brown, Andrew H Talal

**Affiliations:** 1 Department of Biostatistics, SPHHP, University at Buffalo, State University of New York, Kimball Tower, Buffalo, NY, 14214, United States, 1 7168292894; 2 Department of Linguistics, CAS, University at Buffalo, State University of New York, Buffalo, NY, United States; 3 Department of AI and Society, CAS, University at Buffalo, State University of New York, Buffalo, NY, United States; 4 Division of Gastroenterology, Hepatology and Nutrition, JSMBS, University at Buffalo, State University of New York, Buffalo, NY, United States; 5 Department of Public Health, Weill Cornell Medicine, New York, NY, United States

**Keywords:** Large language models, medically underserved populations, opioid use disorder, evidence generation, evaluation, risk, engagement, CREATE framework

## Abstract

Large language model (LLM)–based systems have tremendous potential to improve patient-centered health care, especially for medically underserved populations. However, realizing this potential requires careful consideration of the socio-technical contexts in which LLMs are used. The design of such systems should consider the vulnerabilities of any medically underserved group that the system intends to support, and provide trustworthy evidence for its use. This viewpoint reports the lessons learned from our experience and research with the CREATE (Culture, Respect, Education, Advancement, Trust, and Expertise) framework for engaging multiple stakeholders to guide the integration of LLM-based systems for improved health care delivery. We propose an extension of the traditional hierarchy of evidence model and identify key junction points in clinical workflows at which LLM-based systems can potentially be used to improve patient care. The key messages can be summarized as follows: (1) users of AI technologies, such as LLM-based tools, must be aware of the strengths, limitations, and impact of these technologies on health care delivery workflows and on the quality of generated evidence on which health care recommendations are based; (2) users must also be aware of the impact of data quality on AI outputs and on the evidence generated from the use of these systems; (3) the evidence pyramid provides a framework that facilitates the evaluation of the output generated by AI systems; (4) the CREATE framework facilitates the engagement of a variety of stakeholders. We propose concrete approaches for its validation and implementation; (5) any system designed to support people with opioid use disorder (OUD) needs to consider the overall lack of trust and stigmatizing experiences of this population with the health care system, and we discuss various aspects of the evaluation process necessary to build trust; (6) to discuss research directions that need to be addressed before LLM-based systems can be integrated usefully in patient health care.

## Introduction

Human history has been marked by technological innovation. Generative AI (GenAI), including large language models (LLMs), has resulted in a technological revolution [1]. While LLMs have tremendous technological potential, we, as a society, must be cognizant of the enormous potential social costs of LLMs without deliberate inclusiveness and safeguards. Failure to include the entire population, including medically underserved populations, in the AI technological revolution risks increased wage disparities, reduced productivity, and lower government revenue. Medically underserved populations include geriatric individuals, those with learning disabilities, low-income individuals, homeless individuals, and those with substance use disorder. The effects of the digital divide, conventionally referred to as persistent health care disparities in digital health care access [2], are most acutely felt in these populations. The disparities can lead to exclusion from the use of new technologies, such as AI, especially among underserved populations.

For technological innovations to be acceptable to and accessible to the public, we must consider both social and technical aspects. Examples of sociotechnical innovations in health care include electronic health records (EHRs), communication applications, clinical decision support, and telehealth models. The social aspect of these health care tools involves user workflows and organizational policies surrounding their use, which are influenced by individuals’ level of digital literacy.

To address the social aspects of the deployment of digital technologies, we adapted the CREATE framework proposed by Talal et al [3]. Our adaptation reflects CREATE as (C=culture, R=respect, E=education, A=advancement, T=trust, E=expertise). The CREATE framework outlines the processes to engage stakeholders in the acceptability, feasibility, and usability of digital technologies in various communities. Implementation of new technologies must consider how individuals will engage with the technology. An engagement framework, such as CREATE, is necessary when considering people with opioid use disorder (OUD), a patient population that is typically stigmatized, medically underserved, and difficult to reach [4]. People with OUD enroll in opioid treatment programs (OTPs) for medical and behavioral treatment of addiction recovery. The recovery journey can be difficult, lengthy, and requires dedicated OTP staff involvement to comprehensively manage patients’ progress. New York State was a pioneer in the treatment of OUD [5]. As a result, its procedures for treatment of OUD have been honed over decades. Although in this paper we refer to New York State–based OUD treatment procedures as a use case, substance use disorder treatment requires the collection of extensive clinical information. The lessons learned are transferable to the treatment of substance use disorder and health care more broadly.

In the treatment of OUD, one of the fundamental instruments that inform the creation of treatment plans in OTPs is the psychosocial evaluation form completed within 30 days of admission. The psychosocial form is a comprehensive and detailed history of critical life areas and includes, among other aspects, past and present drug and alcohol use history, prior treatment history, family, legal, and trauma history, and physical and mental health information. The collection of these data and the construction of a patient-centered treatment plan are one of the most important and time-consuming processes of a counselor’s responsibilities. Enhancing the value of the psychosocial instrument by implementing new technologies in the workflows of OTPs has the potential to streamline data collection and mitigate inefficiencies. It might ensure that patient information is collected in a systematic, reproducible, and standardized manner, resulting in high-quality data for use in the development of treatment plans.

The pursuit of high-quality, data-driven evidence enhanced by information generated from complementary sources, such as high-quality qualitative studies, allows individuals to make better choices about health and health care [6]. In the OTP setting, evidence is translated into trust. Before LLM-based systems can be trusted with personal health information, an important objective is to ensure that all stakeholders agree that such systems are error-free, rigorous, and reproducibly generate data-driven evidence. These systems can also be of assistance when collecting data necessary to generate evidence that ultimately leads to improved health outcomes and/or better health care delivery.

The evaluation of technology incorporated into OTP workflows consists of assessing the safety and process compliance of the tools as well as evaluating their effectiveness. Therefore, before embedding LLM-based systems into OTP workflows, a safety evaluation is necessary to avoid and/or correct errors and ensure compliance with institutional metrics and standards (process compliance) [7]. An additional level of evaluation that is required is an assessment of effectiveness. This means that unambiguous and reproducible evidence is obtained that demonstrates improved outcomes. The goal of the evaluation process is a clear demonstration that the embedded technology has a positive impact on the health of individuals and/or the health care delivery process.

A risk-benefit assessment is important to compare potential risks against beneficial outcomes. Identified risks associated with the use of LLM-based systems in OTP workflows include potential biases, hallucinations (ie*,* confabulation by the LLM-based system), and loss of privacy, security, and confidentiality. Benefits include the potential to streamline administrative tasks, resulting in considerable time savings, and improve patient assessment and education. Some of these tasks tolerate the incurrence of small errors that can be corrected by the human interfacing with these systems, while others, such as patient assessment, must be error-free. The development of quantitative risk-benefit measures ensures informed decision-making. Regulatory processes need to ensure the initial safety of the AI tools with continuous monitoring to guarantee quality and adaptability to current circumstances.

The paper explores important social considerations and evidence generation needs when attempting to integrate LLMs into clinical spaces for health care delivery. Recent investigation within the health care arena has focused on LLMs promoting increased patient-centered health care [7-9], partially through the expression of empathy [9]. Therefore, the primary aims of this viewpoint are: (1) to elucidate the strengths and limitations of using AI technologies, such as LLM-based tools, in health care delivery workflows (this aim is articulated in the remainder of the paper). To provide recommendations for potential adoption in clinical scenarios with an emphasis on underserved populations to facilitate patient-centeredness (see sections “What are Recommendations to Improve Applicability and Trust of LLMs to Vulnerable Populations?” and “Considerations for Deployment of AI Systems for Underserved Populations*”*). To discuss the adoption of these systems through the lens of Diffusion of Innovations (DOI) theory [8] (see “LLMs as Socio-Technical Systems: Diffusion of Innovation*”*). (2) To discuss the evaluation of LLM-based systems from multiple aspects (see “Evaluation of LLM-based Systems*”*). (3) To discuss the impact of data quality on AI outputs and evidence generated from these systems; and to discuss the impact of using the generated evidence on health care decision-making (see sections “Use of LLM-based Systems for High-Quality Data Acquisition and Improving Clinical Outcomes,” and “Evidence Generation and Risk Assessment in OTPs”). (4) To present and discuss an extension of the traditional hierarchy of evidence model to include the risks associated with deploying AI-based systems at various stages of data collection, exemplified in the case of OTPs (“Evidence Generation and Risk Assessment in OTPs”). (5) To propose the CREATE framework as a facilitator of the participation of all relevant stakeholders in the development and deployment of AI systems in health care workflows and present several considerations to gain acceptance of LLM-based systems by necessary stakeholders to maximize the likelihood of deployment and use (“Engagement Approaches: CREATE Framework”).

## LLMs: Definition, Advantages, and Limitations

According to the US Congress [9], “Large language models (LLMs) are AI systems that aim to model language, sometimes using millions or billions of parameters.” On a more technical level, LLMs are complex neural networks using the transformer architecture and the attention mechanism proposed in [10]. The word “large” usually refers to the large number of parameters (often billions of parameters) used in training the neural network model to process the language. LLMs require considerable computational resources to be trained. LLMs form the core of LLM-based systems designed to provide various kinds of functionality such as machine translation, summarization, and conversational systems. Multimedia Appendix 1 presents a brief history of natural language processing (NLP) along with a timeline of evolution of the field of NLP (Figure S1 in Multimedia Appendix 1), and Multimedia Appendix 2 briefly discusses details associated with LLMs and applications in health care [11-14].

With newer applications of LLMs emerging every day, it is imperative to be informed of the limitations of these systems as well. Several technical and social challenges are associated with deploying any LLM-based system and their use context. The technical challenges of such systems are discussed in detail in this article from the perspective of accuracy of the generated output and usability of such systems, which are evaluated primarily on output quality. Unfortunately, their usability is often overlooked in outcome assessment. We emphasize that usability of such systems, including their safety and reliability, is an important consideration because in clinical settings such systems do not exist in isolation but rather as integrated socio-technical systems (STSs) to facilitate patient care (see section “Evaluation of LLM-based Systems”).

Deploying LLM-based tools in health care might also lead to potential social challenges. The authors in [15] describe various risks associated with using LLM-based tools in public health. These risks are even more prominent when serving an underserved population. Given that OTPs function as destigmatizing safe spaces, the implications of introducing LLM-based tools in this environment must be carefully evaluated. The key risks include erecting additional barriers to help-seeking, degradation of patient-provider trust and support systems, missed opportunities to proactively introduce help, and dehumanization and impersonality in care, among others. We provide detailed descriptions of various activities in which LLM-based systems can be used in OTPs while keeping in mind these risks and ensuring that patient-centeredness is not lost.

## LLMs as STSs: DOI

STS is defined as “a complex system that includes both technical and social elements, such as people, technologies, rules, and regulations, that work together to create work processes and products” [16]. The definition of STS immediately points to the fact that LLM-based tools in the context of health care are essentially STSs. The conceptual model for health information technology (HIT) introduced by Sittig and Singh [17] is appropriate for this context. The 8 dimensions of this model are computing infrastructure, clinical content (clinical “data-information-knowledge continuum”), human-computer interface, people, workflow and communication, internal organizational policies, procedures and culture, and external rules and regulations. This conceptual model is appropriate for an LLM-based system as well. In the context of an LLM-based system, the technical component is represented by the computing infrastructure, the model architecture, datasets, and the associated infrastructure required to build such systems. The social component includes system developers, patients, the institution where such a system is deployed, the clinical staff, as well as ethics and any associated regulations. Failure to consider the interdependencies between the different components has the potential to result in the pitfalls described below [18].

1. Framing trap: this trap occurs when a system is deployed without completely understanding the associated social setting and details of the problem the tool aims to solve. The problem formulation needs to incorporate the social context such as people, institutional environments, decision-making processes, and existing regulatory systems.
2. Portability trap: this arises when technologies designed for different social contexts and purposes are transferred to new settings without considering characteristics of the new social setting. This can often lead to harmful consequences.
3. Formalism trap: the evaluation of an STS often requires considering the context in which such systems are used, without which the evaluation of a system is merely mathematical in nature.
4. Ripple effect trap: ripple effects occur due to unintended consequences of introducing new technology to existing social systems (such as an OTP in this case). This can lead to unintended changes in behaviors and values of the existing system.
5. Solutionism trap: the failure to recognize that the solution to a problem might not involve introducing new technology.

The effects of getting stuck in any of these traps in a health care setting are consequential for treatment outcomes and patient health. The successful integration of LLM-based systems into existing settings and workflows depends on understanding the abilities and limitations of the components of the STS, the LLM-based system itself, as well as the social context in which it is deployed. The CREATE framework described later in the paper (Section “Engagement Approaches: CREATE Framework”) emphasizes the social considerations needed to increase the likelihood of full integration of LLM-based systems into OTPs.

To avoid the pitfalls discussed previously, understanding the technical aspects of LLM-based systems alone is insufficient; one must also consider the social system context. We offer insights on the adoption of LLM-based tools in OTPs through the lens of DOI theory. DOI theory has been widely applied to describe the important considerations when introducing any new technology. The adoption of new technology within the health care context is always a multistakeholder approach, which extends beyond the potential technical advantages, considering multiple stakeholders and defining their role in the adoption process. While DOI describes how an intervention spreads within a setting, CREATE serves as a framework for consideration of the social aspects of intervention adoption. Of particular interest in the OTP setting is its resource-scarce nature and the underserved population it serves, leading to additional trade-off considerations between purported advantages and potential loss of patient-centeredness and straining limited resources. We have described in detail the elements and characteristics of innovation (LLM-based tools) in the context of OTPs in Tables 1 and 2. The DOI theory highlights that the adoption of LLM-based tools is inherently an interplay between the social and the technical components. The aspects of trust and risk associated with such STS are discussed in a later section in this paper.

**Table 1.** The elements of innovation adapted to large language model (LLM)–based tools in the context of opioid treatment programs.

Attribute	Entity	Relevance
Innovation	LLM-based tools to improve and aid in patient outcomes.	Focus on patient safety in a medically underserved population. Efficiency and decision-making may improve patient outcomes.
Adopters	Providers Government & third-party payers Patients and patient advocates.	Clinical staff are crucial for technological integration into clinical workflows. Regulatory authorities can standardize adoption, maintain care quality, and ensure appropriate system use. Patients and stakeholders provide feedback and ensure effective and ethical use.
Time	AI is in the early stages of adoption in the OTP^a^ setting.	Patient safety is crucial for medically underserved populations. Patients face stigma and other barriers. Ensure patient safety through maintaining privacy & data confidentiality, without harm.
Communication	System Development Financial Engagement Care Delivery Shaping Policy	AI system developers with input from regulatory authorities & clinical staff: Effective communication is important during product development. Regulatory and health care providers provide necessary inputs to integrate AI systems into existing workflows. Staff⇔staff, staff⇔patients, patient⇔patient: Communication between staff and patients is essential to build trust in the system. Effectiveness of the systems depends on integration into existing workflows. Payers⇔treatment providers: Reimbursement policies and support are needed for care provided using LLM-based tools.
Social System	Government and Regulatory Authorities Financial Stakeholders Care Delivery Stakeholders Professional Organizations and Advocacy Groups Researchers	Successful development, deployment, and use of socio-technical systems in real-world settings depend on the social system. Engagement with the ecosystem facilitates continuous refinement through feedback. The CREATE^b^ framework (Figure 1) addresses the social aspects when integrating new technology into substance-use treatment programs.

a OTP: Opioid Treatment Program.

b CREATE: Culture, Respect, Education, Advancement, Trust, Expertise.

**Table 2.** Characteristics of innovation for large language model (LLM)–based tools in opioid treatment programs. Adapted from [8].

Attributes	Relevance
Relative advantage	Improved efficiency: LLM-based tools reduce repetitive administrative tasks, freeing providers to focus on clinical tasks. Enhanced decision-making: LLMs can process vast medical texts to aid diagnostic and clinical decision-making. LLM-based systems assisting health care providers can potentially improve care quality and reduce errors. An important consideration is whether these LLM-based systems should be patient-facing or used solely for internal workflows. Potential applications: aid in filling out intake forms. Risks include digital literacy in the OUD^a^ population. Furthermore, a benefit is the destigmatization that can occur. Patients may share sensitive information more comfortably without discrimination and shame occurring during human interactions.
Compatibility	New systems must be compatible with existing workflows. Emphasis on usability. Usability focuses on learnability, efficiency, memorability, error prevention & recovery, and user satisfaction. Companies are integrating LLM-based solutions into EHR^b^ systems for various uses [19].
Complexity	Applications can be grouped into one of the three groups: Low complexity Simple tasks for patient communication are low complexity and have minimal associated risk. Medium Complexity LLM-based systems used for patient education, such as answering queries, providing instructions, and offering health education. Associated risk can be mitigated potentially through grounding the responses on a knowledge base. The system should not be detrimental to human health. High Complexity Examples include diagnostic and decision-making support. LLM-based systems must ensure safety, build trust, ensure usability, risk education, and effective training for staff and patients for seamless integration.
Trialability	Most LLM-based applications are not presently implemented in OTPs^c^, indicating an opportunity for adoption in this domain. Predeployment trials are essential before deploying any AI system, especially in health care, where errors are unacceptable. Community partnerships (eg, between OTPs and research universities) can facilitate these trials. Active stakeholder engagement (eg, site visits and stakeholder engagement) is needed for extending, addressing, and facilitating large-scale implementation.
Observability	Current implementations of LLM-based systems directed toward the OUD population or OTPs do not exist. Evidence from other areas in medicine highlights the potential of LLM-based tools in improved patient-doctor communication [20], generating patient-friendly and accurate notes [20], enhanced diagnostic accuracy in pulmonology and endocrinology [21], pathology report explanation [22], medical exam recommendations and diagnosis [23], clinical decision support systems [24].

a OUD: opioid use disorder.

b EHR, electronic health record.

c OTP: Opioid Treatment Program.

**Figure 1.**
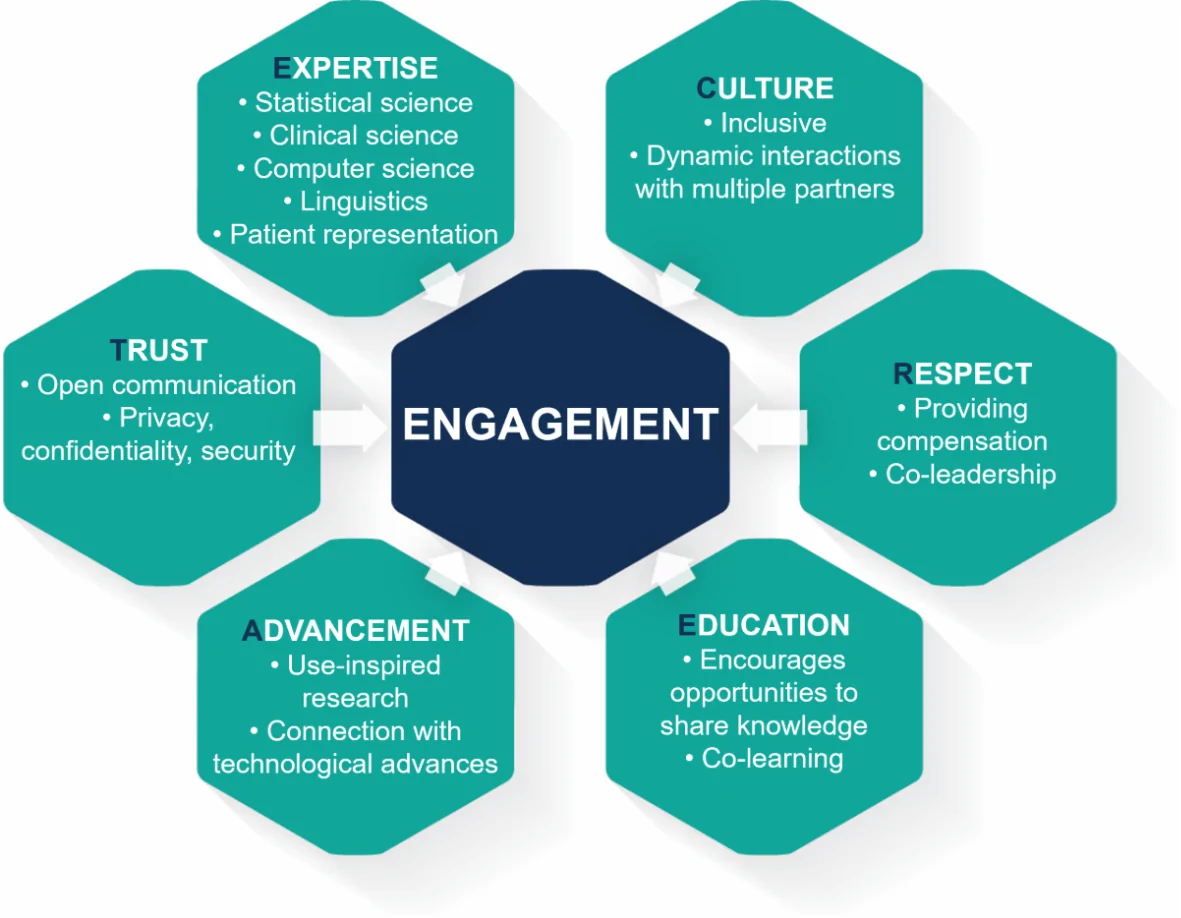
CREATE (C=culture, *R*=respect, E=education, A=advancement, T=trust, E=expertise) framework for engaging stakeholders in the integration of AI within health care.

## Engagement Approaches: CREATE Framework

To guide engagement of stakeholders with LLM-based systems, we extended the CREATE framework developed in [3] (Figure 1). The CREATE framework outlines the processes to address the social aspects of technological integration into a community. Elucidating clinical workflows for patient care facilitates understanding of the OTP culture. Respect for OTP staff and patients is manifested by incentives, avoiding stigmatizing language, and through co-leadership decisions. Education about technology, addressing anxiety through knowledge, and co-learning between patients and staff promote a respectful attitude toward technology. Education also promotes technological advancement through invention and the application of new tools. Promoting trust among patients and staff in LLM-based tools is required for deployment. LLM use is enhanced by ensuring privacy, confidentiality, and security of the technology. Successful LLM deployment and use require expertise in multiple disciplines, and the CREATE framework can address the social aspects of technological deployment in underserved communities. Considerations for evaluation matrices of each of the 6 CREATE framework domains are illustrated in Table S1 in Multimedia Appendix 3. Consideration of the CREATE framework domains, as judged against conventional implementation frameworks, is listed in Table S2 in Multimedia Appendix 3.

As a major funder of comparative effectiveness research, the Patient-Centered Outcomes Research Institute (PCORI) has developed 6 foundational aspects of research partnerships. Table 3 illustrates how the CREATE framework aligns with PCORI’s 6 foundational expectations for partnerships in research [25].

**Table 3.** Foundational aspects of research partnership.

Category	Definition	Relevance to CREATE^a^ framework
Representative involvement	Inclusion of partners and organizations that reflect a range of affected patients and communities.	Multidomain expertise is required for engagement success and promoting advancement.
Build capacity to work as a team	Identify strengths and obstacles to engagement; provide education to address obstacles.	Understanding culture and education can identify engagement opportunities; obstacles are overcome with education.
Early and ongoing engagement	Responsible party engaged throughout the technology lifecycle	Understanding culture illustrates how and where to engage.
Ongoing review and assessment of engagement	Perform continuous assessment and evaluation of successes and failures.	CREATE provides an overview of how engagement might be bolstered or modified
Meaningful inclusion of partners in decision-making	Use approaches to include multiple partners in decision-making	Principle promotes co-learning and multidomain expertise.
Dedicated funds for engagement and partner compensation	Resources to compensate for time and effort.	Funds can promote respect and trust as CREATE components.

a CREATE: C=culture, R=respect, E=education, A=advancement, T=trust, E=expertise, explains social aspects of technology deployment in a community.

## What Are Some of the Functions of LLM-Based Systems in OTPs?

LLM-based systems have shown good performance in several use cases. Some examples include responding to prevention questions from patients in cardiology [26], hip replacement [27], and radiology report findings [28]. Novel experiments integrating LLMs as clinical decision support tools are needed to evaluate their effect on outcomes, productivity, and patient satisfaction [29]. Existing literature has evaluated LLMs’ abilities to respond to portal patient messages [30], generate discharge summaries [31], generate structured templates for radiology [28], and support the management of breast cancer tumor boards [32]. Furthermore, LLM-based systems have been successfully implemented in diagnostic image processing and clinical decision support [33]. They play a role in social media monitoring and track trends in OUD that correlate with real-time morbidity and mortality reporting [34]. AI can also be useful in the development of medical guidelines [35] and in the construction of systematic reviews [36].

Part of the challenge of integrating LLM-based systems into clinical workflows is that minor changes in the inputs produce unpredictable outputs [29]. Furthermore, the technology is evolving rapidly, and as it becomes more widespread, a potential issue is overreliance on its output while negating its limitations and biases. As an alternative to AI in the application to health care, the American College of Physicians recommends the term “augmented intelligence” when referring to the role of AI in clinical decision-making. The objective is to promote the concept that human intelligence continues to be central to the clinical decision-making process even when integrating AI, and that AI is a tool to assist clinicians [37,38].

LLM-based systems can be used to address the digital divide. We can use knowledge generation and educational functionalities to enhance digital literacy among patients. LLM-based systems can also be used to combine data on a particular patient within an electronic medical record and to support prior authorizations for medications. In these situations, it would be helpful to have the human in the loop [39] to be able to verify information. Human-in-the-loop (HITL) refers to the integration of an expert into a machine learning or AI workflow to improve the quality of the system’s intended outcomes. In the health care context, expert knowledge and experience are essential, requiring the augmentation of skilled experts, rather than subordinating their skills. Similar to HITL, the terminology “Doctor-in-the-loop” has also been coined [40]. Several works [41] have explored the incorporation of expert knowledge to improve the trustworthiness of AI system–based workflows. In health care, transparency is one of the key requirements for LLM-based systems, which can then support the HITL framework to increase trust in the system [42].

Appropriate LLM-based systems might promote patient-centeredness. LLMs can de-stigmatize language and can suggest alternative language choices. The goal is to promote more empathetic language among health care providers and in public health messaging [43]. LLMs can potentially play an important role in patient and staff education. They can develop culturally and linguistically relevant educational materials, potentially improving patient understanding, medication adherence, and trust in providers [44]. LLMs, in combination with structured EHR data, can develop models that predict treatment attrition, enabling targeted interventions [45]. They may have a potential role in OTP staff training and simulation. LLM-driven virtual patient agents can simulate realistic patient-provider interactions, offering a controlled and ethical environment for training OTP staff in therapeutic dialogue strategies for addiction recovery [46]. The goal is to promote LLM-directed patient-centered messaging and education.

## Potential Use of LLM-Based System in Treatment Plan Development and Implementation

As presently performed, the generation of a client’s treatment plan is a relatively lengthy and laborious process as described in Figure 2.1. When a patient is admitted to an OTP in New York State, the first point of contact is an initial phone screen that gathers basic demographic information and assesses patient eligibility for treatment of OUD. The next step is an admission eligibility assessment by a physician or advanced practice provider. Once the patient is admitted to the OTP, the clinician completes an admission form that includes more extensive information on drug use, residential situation, family history, and information on employment and education. The patient is then assigned to a primary counselor within 24 hours who schedules an initial session to obtain the psychosocial form, which must be completed within 30 days of admission (email, personal communication, Office of Addiction Services and Supports [OASAS], New York, March 31, 2026).

**Figure 2.**
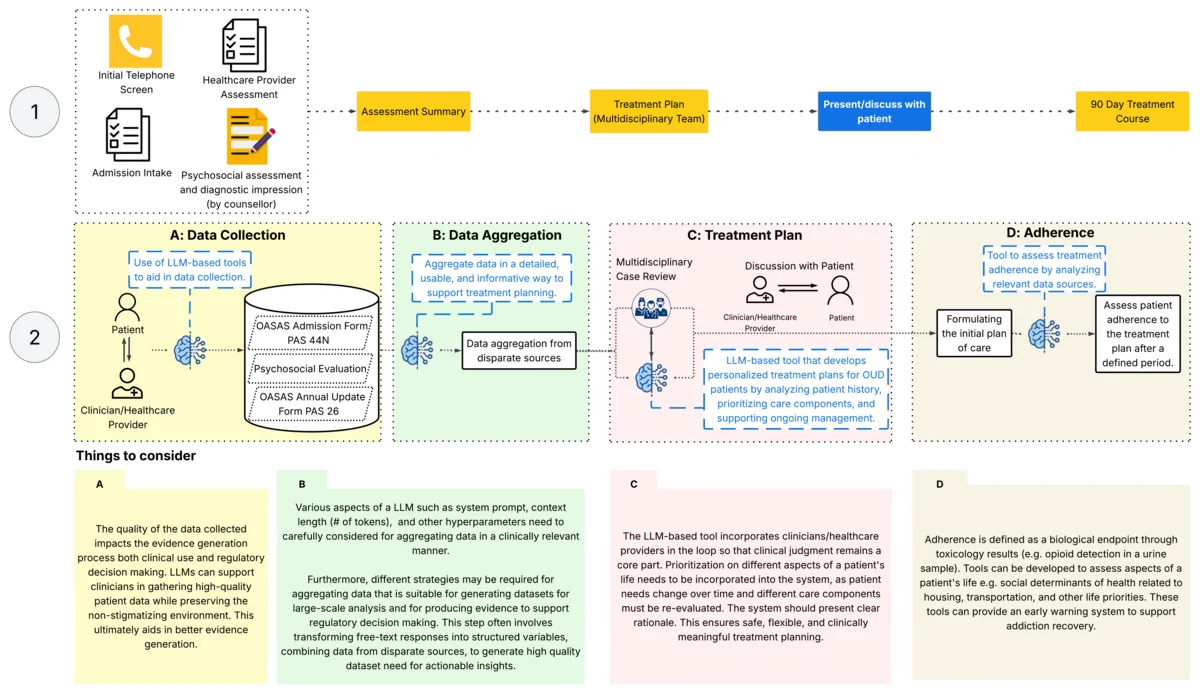
(1) Inputs to and Development of Treatment Plan: The steps for the development of the treatment plan are outlined in the text. Yellow highlight indicates opportunities for task coordination with large language models. (2) Extension of Figure 2.1, which highlights the key junctures where AI-based systems can be leveraged in the day-to-day Opioid Treatment Program workflow. Additionally, we have emphasized certain aspects of AI-based systems that need to be considered before such systems are implemented in real-world settings. For each distinct phase, we illustrate the role of the AI-based system in the blue box. The interaction of various components of AI-enhanced Opioid Treatment Programs is depicted using arrows. Each stage of the workflow can have different extents of patient centeredness embedded within it depending on the patient needs. LLM: large language model; OASAS: Office of Addiction Services and Supports.

Once the psychosocial form has been completed, data are aggregated from the 4 sources illustrated in Figure 2.1 to develop an assessment summary. Upon completion of the assessment summary, the multidisciplinary team meets to develop a treatment plan. The next step is to present and discuss the treatment plan with the patient. Following the discussion with the patient, an initial 90-day treatment course is implemented.

There are several steps at which LLM-based systems can be involved during the creation and implementation of the treatment plan, as illustrated in Figure 2.2. LLM-based systems can be integrated into the data collection stage to facilitate the collection of higher-quality data to inform patient care. Additionally, LLM-based systems can be used to aggregate the collected data from different sources with varied structures to develop treatment plans that consider the unique circumstances of a patient, prioritizing specific aspects of the treatment and supporting ongoing addiction management. Finally, such systems can be used to aid treatment adherence, which is known to be a difficult challenge in the case of addiction recovery. Important considerations for integration of LLM-based systems in different areas of the workflow are also presented.

Furthermore, an empathetic LLM-based system could potentially administer the psychosocial assessment and develop its clinical summary. LLMs could also develop the assessment summary through the aggregation of data from multiple sources. In terms of developing a treatment plan, LLMs could prioritize the most important aspects and assess their severity. The multidisciplinary team could ultimately assess and verify the output of the LLM-based system, similar to the HITL approach described previously. Finally, the LLM-based system can support patient adherence to the 90-day treatment plan.

## Use of LLM-Based Systems for High-Quality Data Acquisition and Improving Clinical Outcomes

In the previous section, we have illustrated how LLM-based systems can be used for developing individualized treatment plans for patients with OUD. One of the major components of developing these treatment plans is collecting data using high-quality instruments. The goal of using LLM-based systems is to generate individualized treatment plans dedicated to specific patient types and reduce associated errors.

From a higher level of abstraction, an important research interest is how LLM-based systems or LLMs can be used for processing vast amounts of data to inform better patient care. OTPs collect massive amounts of textual data consisting of clinical narratives, patient-reported information, and other records. LLMs can be used for converting free text into structured datasets, performing data extraction, and other data processing tasks. These procedures broadly aid in dataset generation that informs care about specific populations.

Table 4 lists the various proposed applications of LLM-based systems along with associated evidence of their implementation in health care situations and briefly indicates whether HITL might be required for oversight.

**Table 4.** Various proposed applications of large language model (LLM)–based systems in health care along with associated evidence and need for human-in-the-loop oversight.

Proposed application using LLM-based system	Evidence	HITL^a^ required (Yes or no)
Patient portal messaging	[30]	Scheduling does not require HITL. Health care inquiries need HITL validation.
Clinical decision support systems	[47,48]	Yes
Destigmatizing language and suggesting empathetic alternatives	[43,49]	Yes
Patient and staff education using relevant materials	[11]	Partial. HITL is needed for verification of information accuracy and updating of knowledge base.
Predicting treatment, attrition & targeted interventions	[45]	Yes
Patient simulation	[46]	Partial. HITL is needed for updating and verification.
Administering intake questionnaires	[50]	Yes
Generating clinical assessment summaries	[51]	Yes
Free text to structured report generation	[52]	Yes
Developing treatment plans	[53,54]	Yes
Supporting patient adherence to treatment plans	[55]	Dependent on the magnitude of the task. For simple and routine verification of adherence, HITL is not required.
Enhancing digital literacy	Proposed	Partial
Data aggregation from various sources to produce a new dataset	Proposed	Yes

a HITL: human-in-the-loop.

## Evaluation of LLM-Based Systems

Prior to the integration of any LLM-based systems, or rather any system in the health care context, understanding associated challenges and evaluating risks vs benefits is of utmost importance. These challenges impact the reliability of generated output, and consequently the quality of evidence and hence need to be addressed to ensure safety and trust. A summary of challenges in the health care context is given in Table 5.

The challenges discussed in this section necessitate a robust evaluation strategy considering multiple aspects described in the next section.

**Table 5.** Key challenges, descriptions, and potential mitigation strategies for use of large language model (LLM)–based systems in health care.

Challenges	Description	Mitigation and solution
Hallucinations	Producing outputs that are factually incorrect and misleading [56]. Divided into factual (discrepancy between generated output and real-world information) or faithfulness (discrepancy between generated output and provided instructions). These issues arise due to training data with outdated or false information [57]. Validation fatigue and optimization for answers that look correct make detecting hallucinations difficult [58].	Retrieval augmented generation (RAG), where responses are based on external knowledge bases [59]; several techniques described in [56].
Sycophancy	The tendency of LLMs to excessively agree with users [60].	Implementing guardrails, ie, controlling the output of an LLM to respect some human-imposed constraints [61].
Black-box nature	Poses a significant challenge to explainability of the model outputs.	Potential techniques for improving explainability for LLMs can be found in [62].
Data protection	Systems must strictly adhere to global data protection laws to ensure patient privacy and confidentiality are not compromised.	Strict adherence to laws like Health Insurance Portability and Accountability Act (HIPAA) and General Data Protection Regulation (GDPR).
Nondeterminism	Poses a serious threat to reproducibility. Arises primarily due to a combination of floating-point non-associativity and parallel execution [63].	Quantifying the uncertainty associated with LLM-based tools is essential for building trust and consequently their large-scale adoption and use in health care. Potential research in these directions is discussed in [64-66].

## Multidimensional Evaluation of LLM-Based Systems

### Overview

The American Medical Informatics Association (AMIA) recommends evaluation of AI systems in health care through multiple lenses, namely technical performance, health impact (effectiveness), and usability and workflows (operational efficiency) [67]. This emphasizes that evaluation of LLM-based systems must not be based on technical aspects alone; rather, it must account for the STS in which it operates.

We describe these evaluation aspects below for clarity. We would like to note that these aspects are not isolated; rather, they are interdependent. For the purposes of this article, we consider usability under technical evaluation, but it is an overlapping concept that links the technical implementation of the system with its adoption and hence effectiveness.

### Technical Evaluation

The technical evaluation should consider the following factors affecting LLM outputs:

1. Parameters: LLM outputs depend on several user-defined parameters such as temperature, top-p, frequency penalty, presence penalty, and thinking or reasoning levels, which in turn affect the output. For instance, how does a set of parameters, for example, θ=[temperature, thinking level, frequency penalty], impact the output O? Understanding the impact of these parameters is an important consideration for our work. Prompting also plays an important role in the output of LLM-based systems. Various prompting techniques, such as described in [68], need to be investigated depending on the task.
2. Repeatability and faithfulness: few works [69] have explored concepts such as:
  - Semantic repeatability: the consistency of LLM responses across repeated runs under identical conditions.
  - Semantic reproducibility: the consistency across runs under different conditions.
3. HITL validation approaches are needed to ensure that evidence generated by LLM-based systems is accurate.
4. Usability evaluation: the definition of usability as given in ISO 9241‐11 is “The extent to which a product can be used by specified users to achieve specified goals with effectiveness, efficiency, and satisfaction in a specified context of use” [70]. Usability evaluation of LLM-based systems is needed to understand whether the tool is practical, intuitive, and readily adoptable by clinical staff without introducing significant workflow hurdles or cognitive burdens. Several usability assessment techniques are available such as the Nielsen usability checklist, questionnaires, interviews, and observational studies, the System Usability Scale, usability testing, cognitive walkthrough, etc. Dehghani et al [71] present a review of methods used in usability evaluation of hospital information systems.
5. Computational reproducibility for provenance: computational reproducibility is defined [72] as ensuring that the same results using the exact same raw materials, computational steps, codes, and conditions as the original analysis can be obtained, and is the foundation for system provenance. In the context of LLM-based health care systems, provenance requires recording and tracking the entire lifecycle of an input (eg, patient data) and its corresponding output (eg, a treatment recommendation). Provenance in case of health care systems is extremely important for legal and regulatory accountability as well as for ensuring that the system behaves as intended. LLM-based systems should be able to trace back how a certain output was generated. LLM-based systems should be developed while keeping this in mind, and in turn, must be evaluated based on it as well.
6. Postdeployment monitoring: an analogue of postdeployment monitoring is postmarketing surveillance of medical products, which emphasizes the need to monitor medical products once they have been placed on the market following rigorous clinical trials. Similarly, to understand the performance of an LLM-based system and address potential shortcomings in real-world settings, postdeployment monitoring is of crucial importance to ensure that the system supports its intended users for its intended purpose. When LLM-based systems are deployed as a component of clinical workflows, several additional mechanisms are required simultaneously to ensure their holistic integration. Several frameworks have been proposed emphasizing various aspects of AI governance [73-75]. Regarding postdeployment monitoring, Keyes et al [73] propose 3 complementary principles: system integrity, performance, and impact. System integrity addresses information technology components, such as maximizing system uptime, detecting runtime errors, and mitigating unintended consequences. Activities essential for performance monitoring include logging, auditing, evaluating bias, accuracy, predictability, transparency, and version control. Impact monitoring focuses on evaluating the value of the system and may lead to recalibration, rollback, suspension, or retirement when deployed systems no longer perform adequately. Additional administrative and institutional policies are needed for incident management and response. El Arab et al [75] found that trustworthy AI implementation in health care settings is increasingly associated with continuous “lifecycle governance” as compared to predeployment validation only. Lifecycle governance is also a cornerstone of continuous quality improvement, which represents iterative improvement of “processes, safety, and patient care” [76]. However, there is a lack of formal consensus, and practical challenges remain regarding metric selection, thresholds for action, review frequency, corrective response, and institutional accountability. These activities align with guidance from World Health Organization (WHO), which recommends that governments should introduce mandatory postdeployment auditing and impact assessments [77].

Additional aspects of technical evaluation should also be considered, keeping in mind the context in which the system is deployed. For example, whether the output adheres to specific guardrails enforced in the system.

### Operational Efficiency (Workflows)

The objective for implementing any LLM-based system in a health care setting is to improve operational efficiency without compromising patient care. Given the administrative workload in OTPs, the system must not introduce additional cognitive overload as discussed in the usability evaluation. The LLM-based system should be evaluated on its ability to streamline workflows. Efficiency in this context is measured by how well the tool reduces the time and cognitive resources of clinical staff in accomplishing a task as compared to currently used solutions (baseline).

### Effectiveness

The implementation of any new system must prioritize and enhance patient care and/or health care delivery. In an OTP setting where LLM-based systems could be used to assist or potentially develop treatment plans, an error-free, highly accurate, and efficient performance is a requirement.

Evaluating health care delivery workflows involves analyzing clinical and administrative processes with the goal of identifying bottlenecks, reducing errors, and ultimately enhancing clinical care. The goal is to evaluate the impact of LLM-based workflows on health care outcomes. Several methods that evaluate technology-enhanced workflows, for example, the use of EHRs, exist in the literature. In the OTP context, if we desire to compare the effectiveness of LLM-based versus standard workflows, we could propose a noninferiority clinical trial using a biological outcome, for example, quantity of opioids measured in urine (ie, toxicology results). In designing trials of clinical effectiveness, one needs to understand the clinical workflows where LLM-based systems will be implemented and the factors beyond the technical capabilities of LLMs to bridge the gaps between implementation and adoption by OTP staff.

## Evidence Generation and Risk Assessment in OTPs

Given the potential benefits and risks of implementing LLM-based systems in health care programs, decisions about their adoption must be guided by a balanced assessment of both dimensions. Beyond the general concerns discussed in prior sections, levels of risk of LLM integration in a health care program should be assessed critically based on the function of the model and the type of input data. For a better visualization of the benefits and risks aspects of LLM-based systems, the pyramid model presented in Figure 3 illustrates the central rationale for LLM integration in OTPs. The deeper the integration of an LLM model with more clinically dedicated tasks and the richer the data sources, the higher the level of evidence obtained, and consequently, the higher the risk associated with LLM-based systems (Figure 3).

Telephone screening is the foundation of the pyramid, which typically occurs before formal OTP admission. This interaction involves acquiring basic demographic information and assessing patient eligibility for OUD treatment. The LLM-based system functions (eg, summarizing call transcripts, extracting key variables, and generating reminders for follow-up) work with the lowest level of input. As a result, errors from LLM-based systems at this stage rarely affect immediate clinical decisions, so the risk is minimal. Thus, we obtain the lowest level of evidence generation (–2) in the pyramid model. During OTP admission, programs collect patient information on psychosocial forms. The pyramid illustrates that, at each subsequent level of risk, the complexity of clinical information increases. Simultaneously, the risks and consequences such as a breach become increasingly costly.

**Figure 3.**
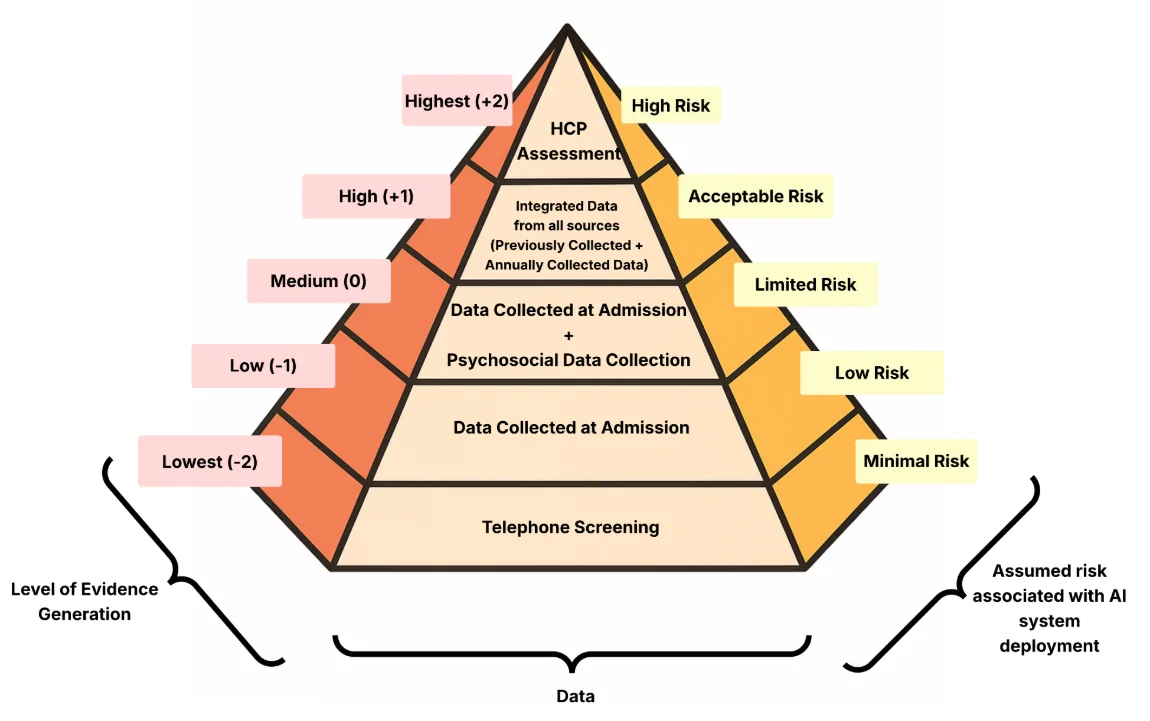
A multifaceted pyramid of data, evidence generation, and risk associated with AI systems in an Opioid Treatment Program setting. The front face, representing data, highlights the hierarchy of data available at Opioid Treatment Programs for opioid use disorder treatment. When an opioid use disorder patient is first admitted, a screening phone call is conducted, acquiring preliminary data (lowest quality). Gradually, as the patient is incorporated into the Opioid Treatment Program, collection and aggregation of additional sources of data take place, which leads to a higher quality of data. Corresponding to each level of data quality, there is an associated level of evidence generation (left face). The right face of the pyramid represents the risk associated with deploying AI-based systems in each stage of data collection or aggregation, depicted in the front face. HCP: health care provider.

## Regulatory Aspects

Policy frameworks for AI are currently in a state of rapid and continuous change. Regulations and laws written to govern the use of AI neither address the challenges nor the opportunities associated with such systems [77]. In July 2024, the European Union adopted Regulation (EU) 2024/1689, commonly known as the EU Artificial Intelligence Act, thereby establishing the first AI regulation as a comprehensive framework to regulate AI systems [78]. The act includes a risk-based regulatory framework that differentiates obligations for AI developers and deployers according to the level of risk posed by specific systems [78-80], categorizing the risk into unacceptable risk, high risk, transparency risk, and minimal risk. For further information see [78].

In the United States, there are no federal laws dedicated solely to AI. The existing framework relies on a patchwork of device, data privacy (such as the Health Insurance Portability and Accountability Act [HIPAA]), and liability laws. However, the regulatory landscape for AI in the United States is currently undergoing a period of rapid and significant transition [81, 82]. In December 2025, an executive order was issued to foster a more unified national environment for AI by aligning federal and state policies to ensure a supportive regulatory framework for AI innovation [83]. Over a dozen states have enacted laws designed to ensure safeguards and guardrails for the use of AI. For example, New York State’s RAISE Act outlines key AI risks including cybersecurity threats, system failures, criminal contact, weaponization, and critical harm resulting in death or serious injury. For more information, please see the RAISE Act (S.53/A.6453) [84]. The act also has reporting requirements for managing risks including safety protocols, third-party reporting requirements, and auditing procedures. California has also enacted a similar law “Transparency in Frontier Artificial Intelligence Act (SB53)” that is designed to enhance online safety and install guardrails on the development of frontier AI models. Other governments are rapidly developing new laws or regulations or adopting targeted restrictions [77].

Companies are expected to release successively more powerful and capable LLM-based systems in the coming months, which may introduce new benefits but also new regulatory challenges. While these laws and regulations provide general guidelines for the use of AI systems, one must keep them in mind when developing LLM-based systems targeted at underserved populations.

## What Are Recommendations to Improve Applicability and Trust of LLMs to Vulnerable Populations?

While there are many reporting guidelines for the use of AI tools in medicine [85] and many relevant consensus statements [86], it is imperative that the adoption of AI systems is based on robust, reproducible evidence obtained via scientifically sound evaluation processes.

When introducing new technologies to people with OUD, recommendations to build patient-doctor trust include stigma minimization, promotion of empathy in the patient-provider relationship, and engaging community organizations to build bridges with health care providers and institutions. With regard to LLMs, to guard against bias and hallucinations, rigorous benchmarks must evaluate LLM-based systems using medical data and tasks, so that reproducibility can be evaluated and developmental progress tracked [29]. These systems must also be evaluated against, for example, frameworks that have emphasized ethical aspects, such as respecting human values and being inclusive [87] and promoting transparency in AI use [88,89]. Humans should have ultimate oversight to ensure trustworthy AI outputs.

The unknown nature of some AI models, as the fundamental “black box” design of contemporary LLMs [90], can promote distrust among providers [33]. In the development of these tools, clinical safety, effectiveness, and health equity must be top priorities. A continuous improvement feedback mechanism should be used to promote improvements in AI tools and to report adverse events resulting from the use of AI. LLM-based systems may be able to assess for potential biases and inequity in OUD treatment plans across different patient demographics (race, ethnicity, and sex). These systems may also have a potential future role in promoting equitable OUD management decisions [91].

## Considerations for Deployment of AI Systems for Underserved Populations

Policies toward LLM-based system implementation and deployment in health care settings that care for underserved populations have several considerations (Table 6). Consideration of the domains of engagement as well as the different stakeholders is very important. Particularly important are governmental agencies that can encourage adoption of LLMs through financial incentives or reimbursement for their use. Correspondingly, they also have a role in ensuring security, confidentiality, and privacy of LLM-based systems.

**Table 6.** Recommendations for implementation of large language model (LLM)–based systems for underserved populations by stakeholder position.

Domains of engagement	Stakeholders		
	Opioid treatment program (patients, leadership, administrators, and staff)	Academia and industry	Government
Patient care workflows	OTP^a^ staff explore the effectiveness of LLMs as tools to promote patient engagement.	Promoting research on areas where LLMs are effective in improving patient health outcomes.	Evaluating aspects of the OASAS^b^ network where LLMs can improve patient health outcomes.
Organizational design	OTP staff participate in education on the use and dangers of LLMs. OTP staff evaluate and provide feedback on LLM deployment. OTP staff evaluate whether LLMs can safely reduce staff loads in OTP workflows.	Understanding OTP culture and community to avoid potential disruptive effects from the uptake of LLMs.	Identify areas where LLMs can safely and effectively assist within OTP workflows. Potentially provide incentives or reimbursement to promote safe LLM use.
Policy	OTP leadership establishes clear guidance on the safe use of LLMs in patient care.	Collaborate to explore how LLM deployment can enhance patient outcomes.	Responsible use of LLMs that is, focus on security, confidentiality, and privacy.

a OTP: Opioid Treatment Program.

b OASAS: Office of Addiction Services and Supports.

## Conclusions and Future Research Directions

Technological innovation is a facet of the human condition and has occurred ever since the evolution of humanity. Consideration of how to increase trust and respect in technology is vital, universal, and extremely important, especially when revolutionary technologies, such as AI, are considered.

In this paper, we discuss the adoption of new technology through the lens of DOI theory and extend the CREATE engagement framework to facilitate the implementation of LLM-based systems into OTP workflows. Future research should seek to evaluate the effectiveness of the CREATE engagement framework as related to LLM deployment. Another area for future investigation is evaluation of implementation outcomes, as would conventionally be assessed using standard implementation frameworks, such as Reach, Effectiveness, Adoption, Implementation, and Maintenance (RE-AIM) [92] or Consolidated Framework for Implementation Research (CFIR) [93]. Evaluation matrices for each of the 6 components of the CREATE framework are listed in the Multimedia Appendix 3 and could serve to highlight specific areas for future research inquiry.

Advances in foundation models, such as multimodal processing, increased planning capabilities, and subsequently the development of autonomous LLM agents with the ability to interact with a wide array of tools, emphasize the need for the CREATE framework even further. “Advancement” drives the adoption of these newer innovations into various aspects of patient care, while “trust” ensures that it is done under rigorous oversight by giving patient safety and confidentiality the highest priority. At the same time, these advances have led to the rise of new challenges, underscoring that “expertise” continues to incorporate the needed skills as new innovations are introduced in LLM-based systems. These developments highlight the indispensable role of human experts in ensuring that LLM-based systems are safely and effectively integrated into clinical workflows.

Olawade et al [94] present a narrative review of HITL AI in health care and discuss associated implementation challenges and future research directions. Along with HITL, further research is needed on human-computer interaction involving AI tools. These tools must be developed in a manner that facilitates the collaboration between humans and AI to build trust, ensure usability, and promote adoption [95]. Additionally, an important research area is understanding the economic implications of deploying these tools in health care [96].

The multifaceted pyramid of data, evidence generation, and risk associated with LLM-based systems illustrates the relationships between the different faces and associated levels of evidence generation. The information present in the collected data is fundamental for high-quality evidence generation. The progression among the different levels of the data face of the pyramid can be interpreted as higher levels in the data hierarchy indicate higher information content, which, subsequently, increases the risk associated with the AI system deployment, while simultaneously increasing the quality of evidence associated with the health care recommendations provided to patients. How to formalize these relationships and their interaction with the CREATE framework remains the topic of a different paper.

While the science behind AI is relatively new, issues to resolve remain and must be satisfactorily addressed to ensure the public’s acceptability of the technology. Recently enacted legislation at the state level to provide guardrails for the use of AI is a step in the right direction, although additional topics remain when considering targeting underserved populations. Multidisciplinary perspectives represented by linguistics, computer science, statistical sciences, and medicine are needed to optimize the health care application of LLMs.

## Supplementary material

10.2196/97131 Multimedia Appendix 1 A brief history of natural language processing and evolution timeline of the field of natural language processing.

10.2196/97131 Multimedia Appendix 2 Details on large language models and brief literature.

10.2196/97131 Multimedia Appendix 3 Evaluation of CREATE framework.

## Abbreviations

AMIA: American Medical Informatics Association
CFIR: Consolidated Framework for Implementation Research
CREATE: Culture, Respect, Education, Advancement, Trust, and Expertise
DOI: diffusion of innovations
EHR: electronic health record
EU: European Union
GenAI: generative AI
HIPAA: Health Insurance Portability and Accountability Act
HIT: health information technology
HITL: human-in-the-loop
LLM: large language model
NLP: natural language processing
OASAS: Office of Addiction Services and Supports
OTP: Opioid Treatment Program
OUD: opioid use disorder
PCORI: Patient-Centered Outcomes Research Institute
RE-AIM: Reach, Effectiveness, Adoption, Implementation, and Maintenance
STS: socio-technical system
WHO: World Health Organization
