# Supplementary material for: From Innovation to Impact: The CREATE Framework as a Blueprint for Large Language Model Adoption in Opioid Treatment Programs
Source: J Med Internet Res. 2026 Oct 7;28:e97131. doi: 10.2196/97131 (PMC13646446; doi:10.2196/97131)
Supplement: Supplementary file 1 [file jmir-v28-e97131-s001.docx]

# A brief history of Natural Language Processing

Natural Language Processing (NLP) is a field within AI that enables machines to comprehend human language. NLP researchers develop tools that allow machines to process and derive meaning from text and speech. Examples of NLP applications include search engines, grammar checking, language translation, and virtual assistants such as Siri. At the core of NLP are language models that assign probabilities expressing the likelihood of co-occurrence of linguistic units such as words, phrases, sentences, based on context. Several papers [1, 2] discuss the history of development of language models.

The field of NLP, now dominated by conversations about and applications of LLMs, was much different prior to 2017. In this section, we attempt to present a brief history of NLP reflecting the continuous evolution of the domain over the past century, of which LLMs are the most recent addition. We have captured the evolution of NLP in Figure 1. The developments in the field are reported in different epochs and within each epoch the main achievements are summarized. The distinct epochs of evolution up to the 1990s are derived from reference [3].

The beginning of NLP was grounded in machine translation, with the objective of designing systems that could translate from one language to another. Early advancements in machine translation dominated from the late 1940s to the late 1960s. From the late 1960s to the late 1970s, the focus was on building semantic information processing systems. This era also saw the beginning of ambitious projects associated with understanding speech. The period from the late 1970s to the late 1980s is known as the “grammatico-logical phase,” where computational grammar research was prominent. This period is also marked by the use of NLP in practical applications. The period from the late 1980s to the late 1990s saw a prominent rise in statistical methods in NLP research. In the 2000–2010 period, the neural network-based approach began to be explored. This period saw the rise of many methods, such as feed-forward neural networks and Latent Dirichlet Allocation, for various tasks. The era from 2010 to 2020 was packed with major breakthroughs such as word embeddings, sequence-to-sequence models, the attention mechanism, the Transformer architecture, and BERT models. The current era since the 2020s is loosely the era of LLMs and associated developments such as autonomous agents and orchestration tools.

Figure S1:Timeline of the evolution of Natural Language Processing (NLP) over the years. From the late 1940s to the late 1990s, the major developments are summarized to simplify the presentation of information. Since 2000, significant milestones have been included with corresponding years.

## References

1.	Anderson A, Jurafsky D, McFarland DA, editors. Towards a Computational History of the ACL: 1980-2008. 2012 July; Jeju Island, Korea: Association for Computational Linguistics.

2.	Wang Z, Chu Z, Doan TV, Ni S, Yang M, Zhang W. History, development, and principles of large language models: an introductory survey. AI and Ethics. 2024 2025/06/01;5(3):1955-71. doi: 10.1007/s43681-024-00583-7.

3.	Jones KS. Natural Language Processing: A Historical Review. In: Zampolli A, Calzolari N, Palmer M, editors. Current Issues in Computational Linguistics: In Honour of Don Walker. Dordrecht: Springer Netherlands; 1994. p. 3-16.
