# Supplementary material for: From Innovation to Impact: The CREATE Framework as a Blueprint for Large Language Model Adoption in Opioid Treatment Programs
Source: J Med Internet Res. 2026 Oct 7;28:e97131. doi: 10.2196/97131 (PMC13646446; doi:10.2196/97131)
Supplement: Supplementary file 2 [file jmir-v28-e97131-s002.docx]

# Details on LLMs and Brief Literature

Recent advancements in Large Language Models (LLMs) allow processing of speech, images, and videos, thus allowing multimodal inputs, transforming LLMs into Large Multimodal Models (LMMs) such as GPT-4 or GPT-5. In this work, we refer to LLM-based systems as tools in which either LLMs or LMMs are integrated to perform certain functions. A particular application of LLM-based systems is conversational systems, such as chatbots (e.g. ChatGPT or Google Gemini). A detailed description of the current research on LLMs can be found in a recent review [1]. A search on Google Scholar with the keywords “large language models in medicine” returns close to 23,200 results underscoring the extent of research that is being pursued in this area. To understand the application of LLM-based systems in the healthcare context, it is important to evaluate the potential advantages and limitations of deploying these systems with a focus on “do no harm” approach.

As LLM-based systems demonstrate the capability to process multimodal data, they can be used in several ways. Particularly in healthcare, several application types, such as patient education (interpreting medical information, providing lifestyle recommendations) [2], medical student education [3], and more complex applications, such as treatment recommendation, have been explored in the literature [4]. The readers can refer to [5] for a more detailed discussion which describes various applications as well as highlights opportunities for LLMs in biomedical applications.

## References

1.	Naveed H, Khan AU, Qiu S, Saqib M, Anwar S, Usman M, et al. A Comprehensive Overview of Large Language Models. ACM Transactions on Intelligent Systems and Technology. 2025;16(5):1-72. doi: 10.1145/3744746.

2.	Aydin S, Karabacak M, Vlachos V, Margetis K. Large language models in patient education: a scoping review of applications in medicine. Front Med (Lausanne). 2024;11:1477898. PMID: 39534227. doi: 10.3389/fmed.2024.1477898.

3.	Zhang Q, Huang Z, Huang Y, Wang G, Zhang R, Yang J, et al. Generative AI in medical education: feasibility and educational value of LLM-generated clinical cases with MCQs. BMC Med Educ. 2025 Oct 27;25(1):1502. PMID: 41146115. doi: 10.1186/s12909-025-08085-8.

4.	Wilhelm TI, Roos J, Kaczmarczyk R. Large Language Models for Therapy Recommendations Across 3 Clinical Specialties: Comparative Study. J Med Internet Res. 2023 Oct 30;25:e49324. PMID: 37902826. doi: 10.2196/49324.

5.	Usuyama N, Wong C, Zhang S, Naumann T, Poon H. Biomedical Natural Language Processing in the Era of Large Language Models. Annu Rev Biomed Data Sci. 2025 Aug;8(1):471-90. PMID: 40245359. doi: 10.1146/annurev-biodatasci-103123-095406.
