# Supplementary material for: From Innovation to Impact: The CREATE Framework as a Blueprint for Large Language Model Adoption in Opioid Treatment Programs
Source: J Med Internet Res. 2026 Oct 7;28:e97131. doi: 10.2196/97131 (PMC13646446; doi:10.2196/97131)
Supplement: Supplementary file 3 [file jmir-v28-e97131-s003.docx]

# Evaluation of CREATE Framework

Table S1: Example evaluation matrices for each CREATE domain

| **Domain** | **Evaluation matrix** |
| --- | --- |
| Culture | - Are they open to new processes? - Do they have an inclusive culture? - Does the venue have experience with technology? |
| Respect | - Providing compensation for engaging with AI? - Practicing co-leadership? |
| Education | - Does the venue encourage opportunities for knowledge sharing? - Do they promote co-learning? |
| Advancement | - Does the venue participate in use-inspired research? - Is there a connection with technological advances? |
| Trust | - Does the venue have open communication? - Are there processes for encouraging staff to discuss complexities? - Does the site have procedures for privacy, confidentiality, and security? |
| Expertise | - Does the venue have expertise in domains required for high-quality data generation, such as information technology, biomedical Informatics, and data science? - Do they have people who can explain the procedures for engagement with AI systems? |

Table S2: Evaluation of the CREATE framework domains as judged against conventional implementation frameworks.

| **Domain** | **Evaluation Definition** | **Evaluation Matrix** |
| --- | --- | --- |
| Adoption | Within a given region, do sites adopt AI technology? | How many sites adopt the intervention? |
| Acceptability | Acceptability is use of the technology | Do sites or patients use the technology? |
| Feasibility | Use of specific features within the technology, accessibility of technology. | - Is there widespread uptake of the technology within a particular setting? - Can staff or patients access the technology? |
| Fidelity | Extent to which intervention is executed as originally planned or intended. | Is the intervention practiced at the venue as originally described? |
| Sustainability | Persistence of an intervention over a given period of time | After one year, what percentage of venues continue to participate in AI technologies? |
| Implementation cost | Amount of money needed to implement the intervention | What is the annual cost to implement the intervention? |
